# Using Manganese‐Enhanced DCE‐MRI to Assess Blood‐Cerebrospinal Fluid Barrier Transport

**DOI:** 10.1002/mrm.70531

**Published:** 2026-07-26

**Authors:** Charith Perera, Zhiping Feng, Shereen Nizari, Phillip Muza, Christina Katsiva, Tammy L. Kalber, Mark F. Lythgoe, Ian F. Harrison, Daniel J. Stuckey, Jack A. Wells

**Affiliations:** ^1^ UCL Centre for Advanced Biomedical Imaging, Division of Medicine University College London London UK

**Keywords:** blood cerebrospinal fluid barrier (BCSFB), brain, cerebrospinal fluid (CSF), choroid plexus (ChP), fluid‐attenuated inversion recovery (FLAIR), gadolinium‐based contrast agents (GBCA), high field (9.4 T), manganese‐enhanced MRI (MEMRI), mouse, preclinical

## Abstract

**Purpose:**

We developed a dynamic, heavily T2‐weighted (hT2w) FLAIR protocol to resolve acute Mn^2+^ transport kinetics, as a proxy for Ca^2+^, across the choroid plexus (ChP)—cerebrospinal fluid (CSF) interface, in vivo. By quantifying intercompartmental Mn^2+^ dynamics, and comparing these with Gd‐DOTA, we sought to characterize blood‐CSF‐barrier (BCSFB)‐mediated tracer transport, combining high in‐plane resolution and 4‐min temporal sampling.

**Methods:**

hT2w‐FLAIR DCE MRI was conducted in anesthetized C57BL/6 mice, receiving intravenous Mn^2+^ or Gd‐DOTA. Semi‐quantitative tracer kinetic modeling permitted regional and inter‐tracer comparisons.

**Results:**

Mn^2+^ rapidly crossed from the vasculature into the CSF, across the ChP, with similar arrival times and time‐to‐peak across compartments. Mn^2+^ delivery into the ChP exhibited a 5.7‐fold steeper uptake rate (*p* = 0.031) and a 5.6‐fold higher peak signal amplitude (*p* = 0.0028) than in the LV‐CSF. In contrast, Gd‐DOTA exhibited a 2.2‐fold shorter ChP plateau duration (*p* = 0.034) and 2.2‐fold faster ChP clearance rate (*p* = 0.0047), compared to Mn^2+^. Within the CSF, there were significant temporal delays with Gd‐DOTA relative to Mn^2+^: a lengthened arrival (eight‐fold, *p* = 0.045) and time‐to‐peak (six‐fold, *p* = 0.024).

**Conclusion:**

Our hT2w‐FLAIR DCE‐MRI approach captured the early BCSFB transport window for Mn^2+^, providing distinct ChP and LV‐CSF kinetics at a higher temporal resolution and with reduced CSF signal contamination compared to prior approaches. These findings highlight that MRI tracer choice strongly shapes measured BCSFB kinetics, and supports the use of Mn^2+^ as a tool for probing compartment‐specific ChP transport.

## 1 Introduction

The choroid plexus (ChP) that forms the blood‐cerebrospinal fluid barrier (BCSFB) is a major transport interface between the systemic circulation and the central nervous system (CNS). The ChP plays a critical role in maintaining CNS ionic homeostasis by facilitating the transport of essential ions. Consistent with a BCSFB‐dominated route, ^45^Ca autoradiography showed a ∼20‐fold greater unidirectional influx into ventricular CSF than into frontal cortex via the blood–brain barrier (BBB) [1]. With calcium governing numerous CNS signaling pathways—ranging from second‐messenger cascades to the generation of depolarization currents—this preferential ChP‐mediated entry route from the vasculature, into the brain, emphasizes the barrier's essential role in preserving the ionic environment [2, 3].

Despite evidence for its essential homeostatic role [4, 5], the understanding of the ChP‐CSF locus in both normal physiology and disease states has been hindered by a lack of noninvasive, readily translatable imaging tools. Previous studies have sought to use gadolinium‐based contrast agents (GBCAs) to assess ChP tracer delivery and distribution [6, 7, 8, 9]. However, the mechanisms underlying GBCA‐induced signal emergence in CSF are poorly understood and believed to reflect nonspecific leakage across the BCSFB, as opposed to defined transport pathways [6, 8, 9, 10].

On the other hand, manganese (Mn^2+^), an established MR‐visible calcium (Ca^2+^) analogue, utilizes Ca^2+^ transport mechanisms to facilitate transport across cells for example, at the BBB and neurons [11, 12]. Accordingly, manganese‐enhanced MRI (MEMRI) has been used to probe neural activity and interrogate barrier transport [12]. This active transport of Mn^2+^ has also been postulated specifically at the ChP by transporters expressed at the epithelium [13, 14, 15]. Notably, several of these transporters are of the same molecular pathway known to conduct Mn^2+^ in other brain regions [16], highlighting the potential of using MEMRI approaches to interrogate ChP transport.

Echoing the findings of ^45^Ca autoradiography studies, prior MEMRI work has reported signal enhancement at the ChP and ventricular CSF [12, 17]. However, most MRI studies investigating tracer ingress at the BCSFB infer ChP activity indirectly from bulk ventricular CSF enhancement, often using single postcontrast acquisitions or delayed imaging timepoints rather than dynamic measurements of tracer kinetics. Consequently, the early delivery phase of contrast transfer—from vascular arrival at the ChP and subsequent appearance in adjacent ventricular CSF—has remained poorly resolved in vivo.

To address this, we developed a dynamic, heavily T2‐weighted (hT2w) dynamic contrast enhanced (DCE)‐MRI protocol to visualize Mn^2+^ transport kinetics across the ChP–CSF interface in vivo as a proxy for calcium transport. This acquisition combines a 4‐min temporal sampling interval with high in‐plane spatial resolution and CSF‐nulled FLAIR contrast to resolve the ChP and adjacent ventricular CSF compartments. This configuration enables measurement of tracer kinetics within the ChP and the adjacent ventricular CSF compartment during the acute delivery phase, rather than inferring ChP activity indirectly from bulk ventricular enhancement.

We hypothesized that Mn^2+^ administration would yield distinct kinetic profiles in the ChP compared to the immediately adjacent lateral ventricle (LV) CSF compartment. Then, given the previous use of GBCAs to investigate CSF tracer ingress [6, 7, 8, 9], we compared Mn^2+^‐induced kinetics with those of the clinically used GBCA, Gadoterate Meglumine (Gd‐DOTA), acquired under identical imaging conditions. Unlike Mn^2+^, which can enter cells via Ca^2+^ transport pathways [13, 14, 15], Gd‐DOTA behaves primarily as an extracellular tracer and does not undergo transporter‐mediated cellular uptake [18]. This comparison allowed us to assess how contrast‐agent properties influence tracer behavior at the BCSFB and to evaluate the capacity of Mn^2+^ to report on ChP‐specific transport mechanisms distinct from GBCA transport.

## 2 Methods

### 2.1 Animal Preparation

All procedures complied with the UK Home Office Act (Scientific Procedures, 1986). Male C57BL/6 mice (14–16 weeks, Charles River UK) were used for MnCl_2_ (*n* = 6) and Gd‐DOTA (*n* = 6) experiments. Isoflurane‐anesthetized mice underwent tail‐vein cannulation (for contrast delivery) and positioning in the MRI cradle (Figure S1). Body temperature (maintained at 37°C ± 0.5°C) and respiration were monitored throughout.

### 2.2 Contrast Agents

Mice received contrast agents (Mn^2+^/GBCA) intravenously. The Mn^2+^ formulation contained 11.25 mM MnCl_2_, 11.25 mM calcium D‐gluconate, and 50 IU mL^−1^ heparin, delivered at 5.56 μL g^−1^ (0.0625 μmol g^−1^ Mn^2+^). Gd‐DOTA (Clariscan, gadoterate meglumine, GE Healthcare, 90 mM with 50 IU mL^−1^ heparin) was administered at the same volume‐to‐weight ratio (0.50 μmol g^−1^). Calcium D‐gluconate supplementation has been shown to mitigate Mn^2+^ cardiotoxicity while preserving MR contrast generation, with demonstrated safety and tolerability in clinical studies [19, 20, 21, 22].

Pilot studies informed GBCA dosing: 11.25 mM Gd‐DTPA (Magnevist, gadopentetate dimeglumine, Bayer Healthcare) produced ∼25% of Mn^2+^ enhancement, whereas 90 mM Gd‐DOTA achieved comparable signal (Video S1, Figure S2).

### 2.3 MRI Protocols

Images were acquired on a 9.4 T Bruker imaging system (BioSpec 94/20 USR) with a horizontal bore and 440 mT/m gradient set with an outer/inner diameter of 205 mm/116 mm, respectively (BioSpec B‐GA 12S2), 86 mm volume coil and a four‐channel array rat brain surface coil (RAPID Biomedical GmbH). Sequences were adapted from the Paravision 360 (version 3.5) software.

#### 2.3.1 Anatomical Reference Scans

As described previously, we utilized a heavily T2‐weighted 3D RARE scan to visualize the location of the ChP within the ventricles (Figure S1a) with a high degree of contrast [23]. A ventricular location rich in ChP was identified on initial images and used to position the final DCE imaging slice.

#### 2.3.2 Calculating IR‐TI for CSF Nulling

CSF‐nulling inversion times were derived from ultra‐long‐TE multi‐TI EPI fits to an inversion‐recovery model [24, 25].

#### 2.3.3 FLAIR‐hT2w Rapid Imaging With Refocused Echoes (RARE) DCE

Scans incorporated the subject‐specific IR‐TI and optimal positioning (Figure S1), and had the following parameters: TE = 76.86 ms, TR = 6000 ms, NA/NR = 5/20, ES = 7.686, RARE factor = 32, Matrix = (256 × 256), FOV = (20 × 20 mm), slice thickness = 0.5 mm, in‐plane resolution = (78 × 78 μm), fat suppression = on, dummy scans = 2, inversion slab = global.

Two baseline repetitions (*t* = 0 and 4 min) were acquired prior to contrast injection (administered at *t* = 6 min). Eighteen additional repetitions were acquired postcontrast (4 min per acquisition; 72 min). Pilot scans were extended to 180 min (Figure S2).

### 2.4 FLAIR Image Analysis

**FIGURE 1:**
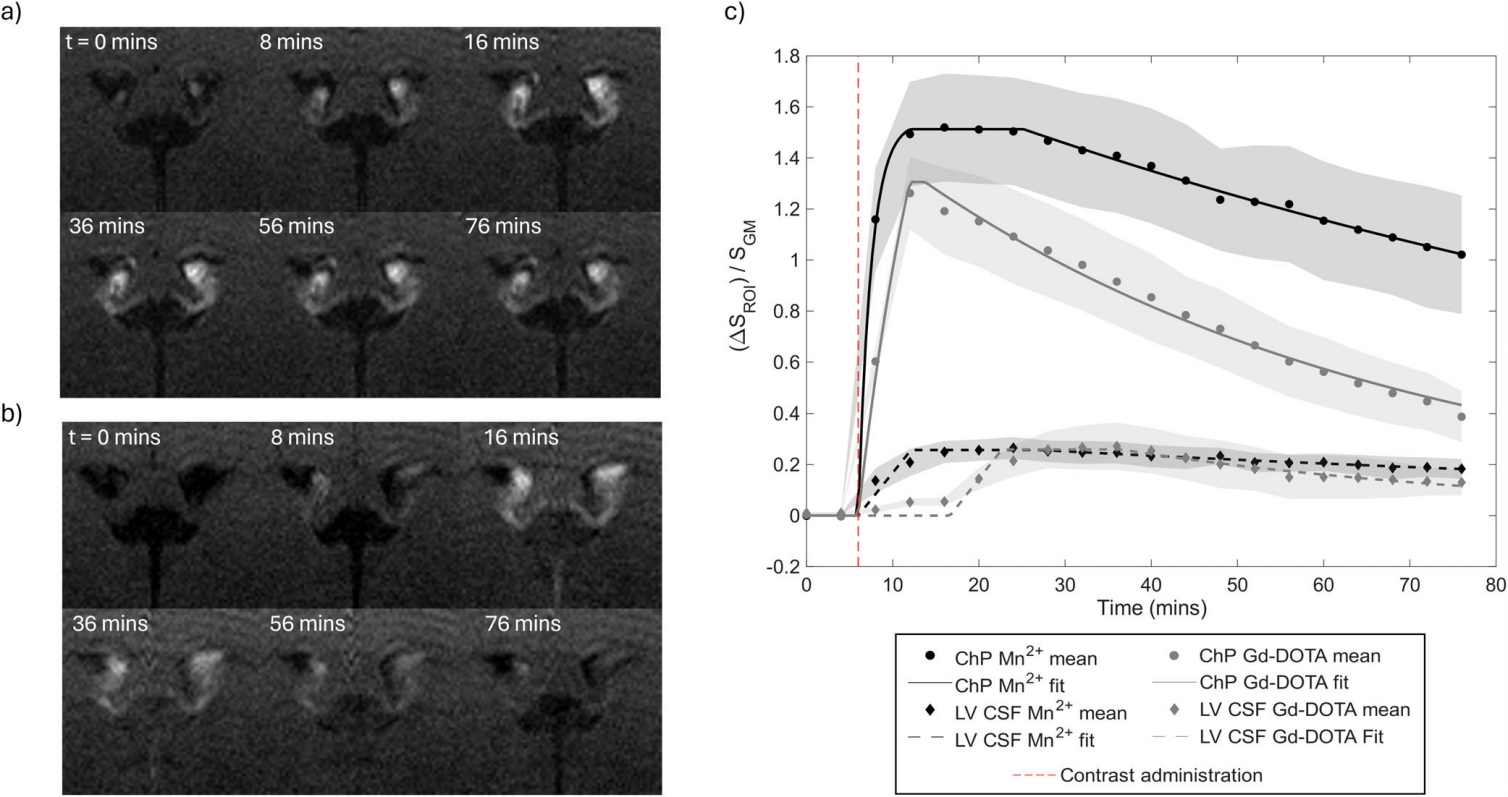
Heavily T2‐weighted FLAIR dynamic contrast‐enhanced MRI data at the choroid plexus‐cerebrospinal fluid locus. Representative image dataset examples (select timepoints) following intravenous administration of (a) Mn^2+^ (*n* = 1), and (b) Gd‐DOTA (*n* = 1). (c) Time course comparison for Mn^2+^ (black) and Gd‐DOTA (gray), displaying the group averaged data points, and model fitting, for the choroid plexus (ChP, solid lines) and lateral ventricular cerebrospinal fluid (LV‐CSF, dashed lines), alongside SEM (*n* = 6 Mn^2+^, *n* = 6 Gd‐DOTA).

Conservative ChP ROIs were manually defined on 3D T2‐weighted anatomical images, excluding adjacent CSF and parenchyma (Figure S1c). Equivalent ROIs were drawn for LV‐CSF and for cortical gray matter (GM) directly above the ventricles (Figure 1c). Mean signal within each ROI (S_ROI_) was extracted per time point and baseline‐corrected (Δ*S*_ROI_). Δ*S*_ROI_ was further normalized to the average baseline signal from the cortical GM ROI (yielding (Δ*S*_ROI_)/*S*_GM_) to compensate for coil‐sensitivity profiles—a critical step given the near—noise‐floor signal in CSF‐suppressed voxels.

### 2.5 Modeling

Tracer uptake kinetics were semi‐quantitatively modeled using a custom four‐phase model that was designed based on observed features from the pilot data (Figure S2): a preinjection baseline, a monoexponential rising phase, a plateau, and an exponential decay.

The normalized signal, [Δ*S*_(ROI)_(*t*)]/*S*_(GM)_, was defined piecewise by six parameters, which were all determined through curve fitting: Smax (plateau amplitude), AT (arrival time), *T*_rise_ (rise duration), *T*_plateau_ (plateau duration), *k*_rise_ (rise rate constant), and *k*_clear_ (clearance rate constant). Further details of this model are detailed in Supplementary Methods in Supporting Information. Time‐to‐peak (TTP) was computed post hoc as TTP = AT + *T*_rise_.

Inter‐regional metrics were derived to compare ChP and LV‐CSF kinetics (i.e., temporal and amplitude differences):

$$\mathrm{\Delta AT}={\mathrm{AT}}_{\mathrm{LV}-\mathrm{CSF}}-{\mathrm{AT}}_{\mathrm{ChP}}$$

$$\mathrm{\Delta TTP}={\mathrm{TTP}}_{\mathrm{LV}-\mathrm{CSF}}-{\mathrm{TTP}}_{\mathrm{ChP}}$$

$$\mathrm{\Delta Smax}={\text{Smax}}_{\mathrm{LV}-\mathrm{CSF}}/{\text{Smax}}_{\mathrm{ChP}}$$

### 2.6 Outcomes and Statistics

Primary outcomes were defined from pilot data (see Figure S2); statistical details are outlined in the Supplementary Methods: Supporting Information.

### 2.7 Ethics Statement

All experiments were conducted in accordance with the European Commission Directive 86/609/EEC (European Convention for the Protection of Vertebrate Animals Used for Experimental and Other Scientific Purposes) and the United Kingdom's Home Office Animals (Scientific Procedures) Act (1986).

## 3 Results

### 3.1 Mn^2+^ to Probe ChP and CSF Compartments

DCE‐MRI experiments were conducted using Mn^2+^ at the ChP–LV–CSF interface. The spatiotemporal evolution of signal is shown in Video S1 and selected time points in Figure 1a. At baseline, CSF suppression was effective, with signal confined to brain tissues such as the ChP and adjacent parenchyma. Group‐averaged kinetic curves for both compartments are shown in Figure 1c.

Following the peak and plateau, a gradual decline in signal was observed in the ChP time‐course, that is, a significant time effect (*F*(16,80) = 55.98, *p* = 1.47 × 10^−27^), and 32% reduction between peak and end points (*p* = 0.00056). Mn^2+^ signal within the LV‐CSF also displayed a significant temporal change (*F*(16,80) = 1.90, *p* = 0.032), with a 33% nonsignificant decrease between peak and final timepoints (*p* = 0.69).

**FIGURE 2:**
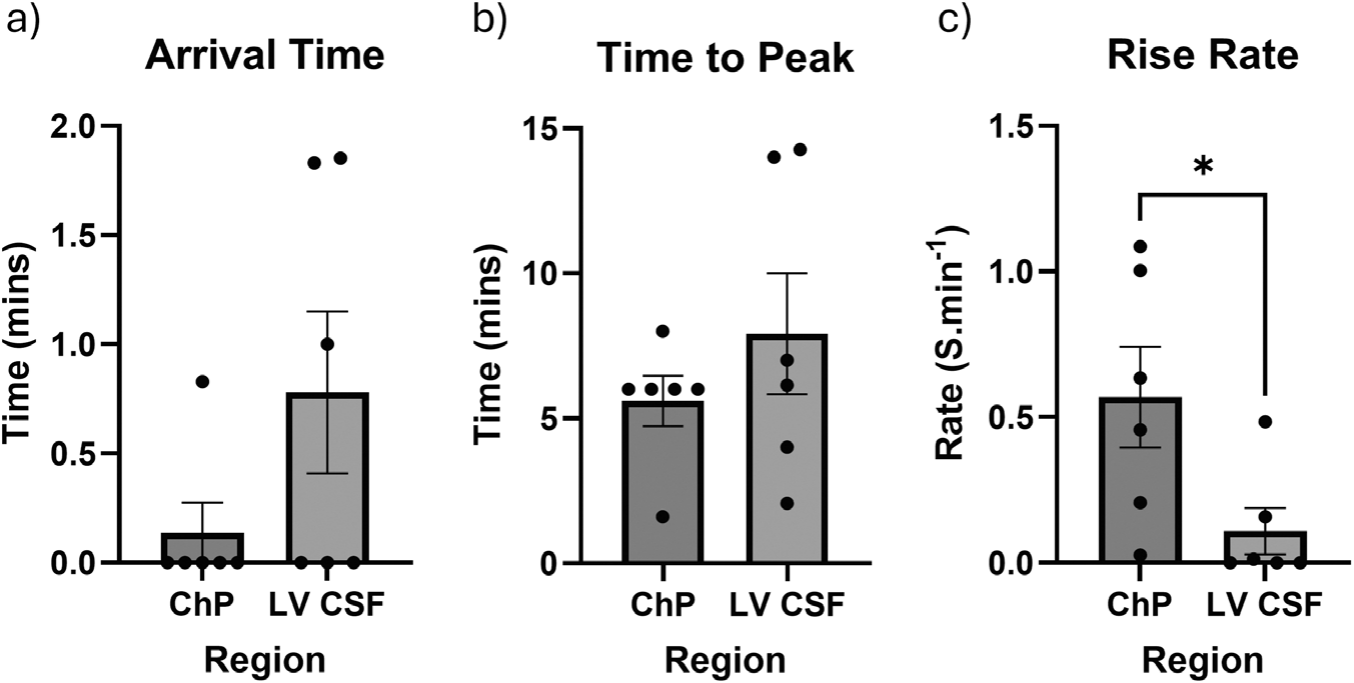
Extracted parameters for heavily T2‐weighted FLAIR following intravenous administration of Mn^2+^. (a) Arrival Time, (b) Time to Peak, (c) Rise Rate. Individual values, mean values, and SEM shown (*n* = 6 Mn^2+^). Asterisks indicate paired ChP–LV‐CSF comparisons: Arrival Time (two‐tailed t‐test), and Time‐to‐Peak and Rise Rate (two‐tailed Wilcoxon signed‐rank tests). * *p* < 0.05; unadjusted.

Examining individually extracted parameters following the administration of Mn^2+^, paired comparisons revealed no significant differences in arrival time (AT) or time‐to‐peak (TTP) between compartments (Figure 2a,b). The ChP exhibited a significantly higher rise rate (5.2×, Figure 2c, *p* = 0.031), Smax (5.6×, *p* = 0.003), and AUC (5.9×, *p* = 0.005) (Table S1). Plateau duration and clearance rate did not differ significantly between compartments (Table S1).

### 3.2 Mn^2+^ Versus Gd‐DOTA at the ChP‐CSF Locus

**FIGURE 3:**
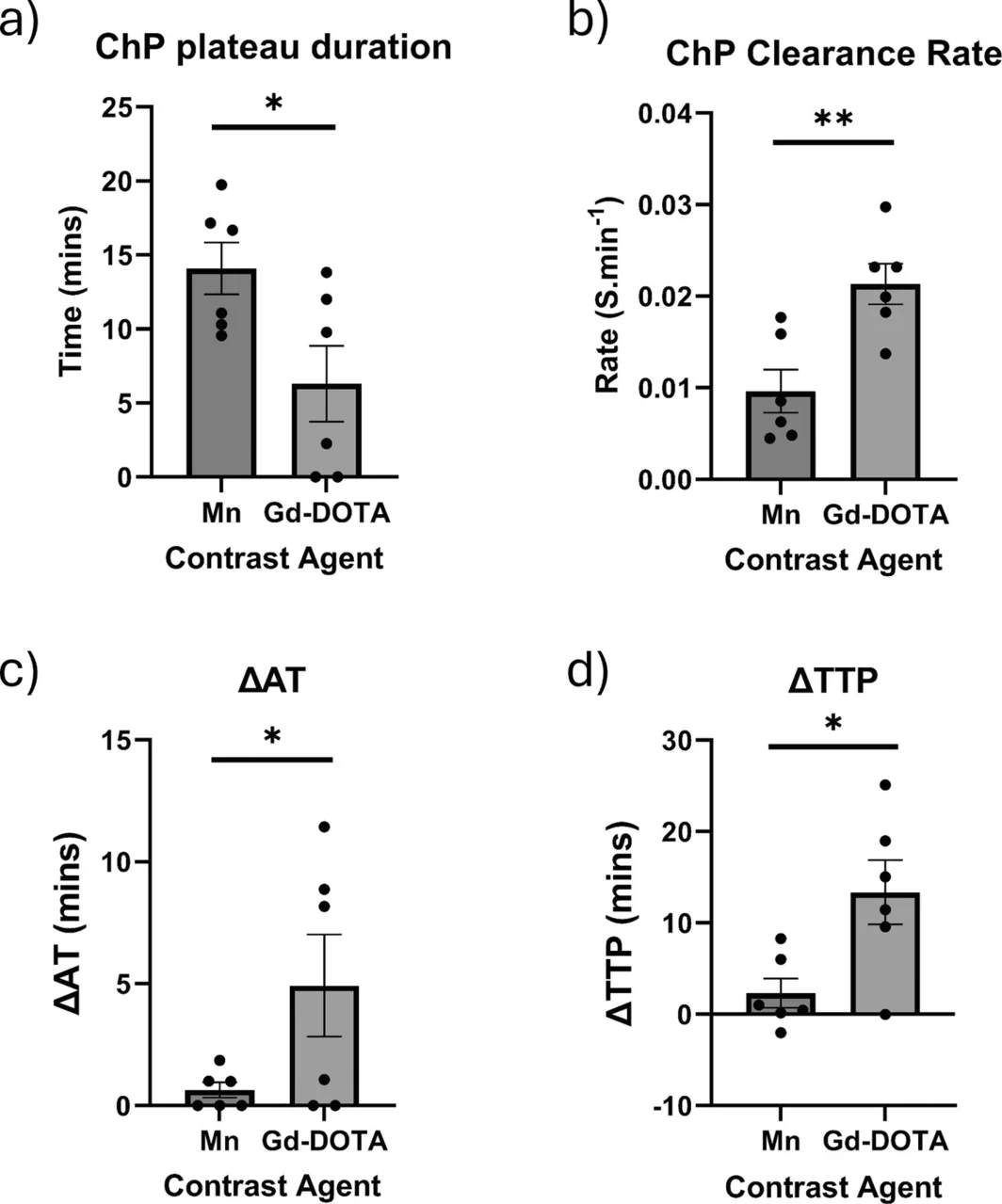
Mn^2+^ vs. Gd‐DOTA contrast‐enhanced MRI data comparison. Individually extracted parameters, alongside respective means and SEMs: (a) ChP plateau duration, (b) ChP clearance rate, (c) difference in arrival time between ChP and LV CSF compartments (ΔAT), and (d) difference in time to peak between ChP and LV CSF compartments (ΔTTP). Individual values, mean values, and SEM shown (*n* = 6 Mn^2+^, *n* = 6 Gd‐DOTA). Asterisks indicate inter‐tracer comparisons, assessed using unpaired two‐tailed Welch's t‐tests. * *p* < 0.05; ** *p* < 0.01; unadjusted.

Using an identical protocol, data collection with Gd‐DOTA allowed direct comparison with Mn^2+^ to assess how each tracer distributes within and clears from the ChP and ventricular CSF compartments (Figure 1b,c, Video S1, Table S2). Group‐averaged ChP time courses (Figure 1c) revealed clear distinctions between tracers, that is, a significant interaction between contrast agent and time (*F*(19,95) = 4.65, *p* = 2.25 × 10^−7^). Consistent with this, Gd‐DOTA exhibited a significantly shorter ChP plateau duration (2.2×, *p* = 0.034) and faster clearance (2.2×, *p* = 0.0047) (Figure 3a,b, Table S2).

Further differences were evident within the LV‐CSF compartment. A two‐way repeated‐measures ANOVA revealed a significant interaction between tracer and time (*F*(19,95) = 2.85, *p* = 0.0004), indicating distinct kinetic profiles for Mn^2+^ and Gd‐DOTA. The mean LV‐CSF AT values were higher for Gd‐DOTA, although this did not reach statistical significance (Table S2). However, when accounting for ChP AT, ΔAT values (Figure 3c) were significantly elevated, with an approximately eight‐fold increase for Gd‐DOTA (*p* = 0.045). Similarly, LV‐CSF TTP (Table S2, *p* = 0.023) and the corresponding inter‐compartment ΔTTP (Figure 3d, *p* = 0.024) were significantly increased, showing an approximately six‐fold elevation for Gd‐DOTA.

All other extracted parameters were not found to be different between tracer treatments for both ChP and LV‐CSF compartments (Table S2).

**FIGURE 4:**
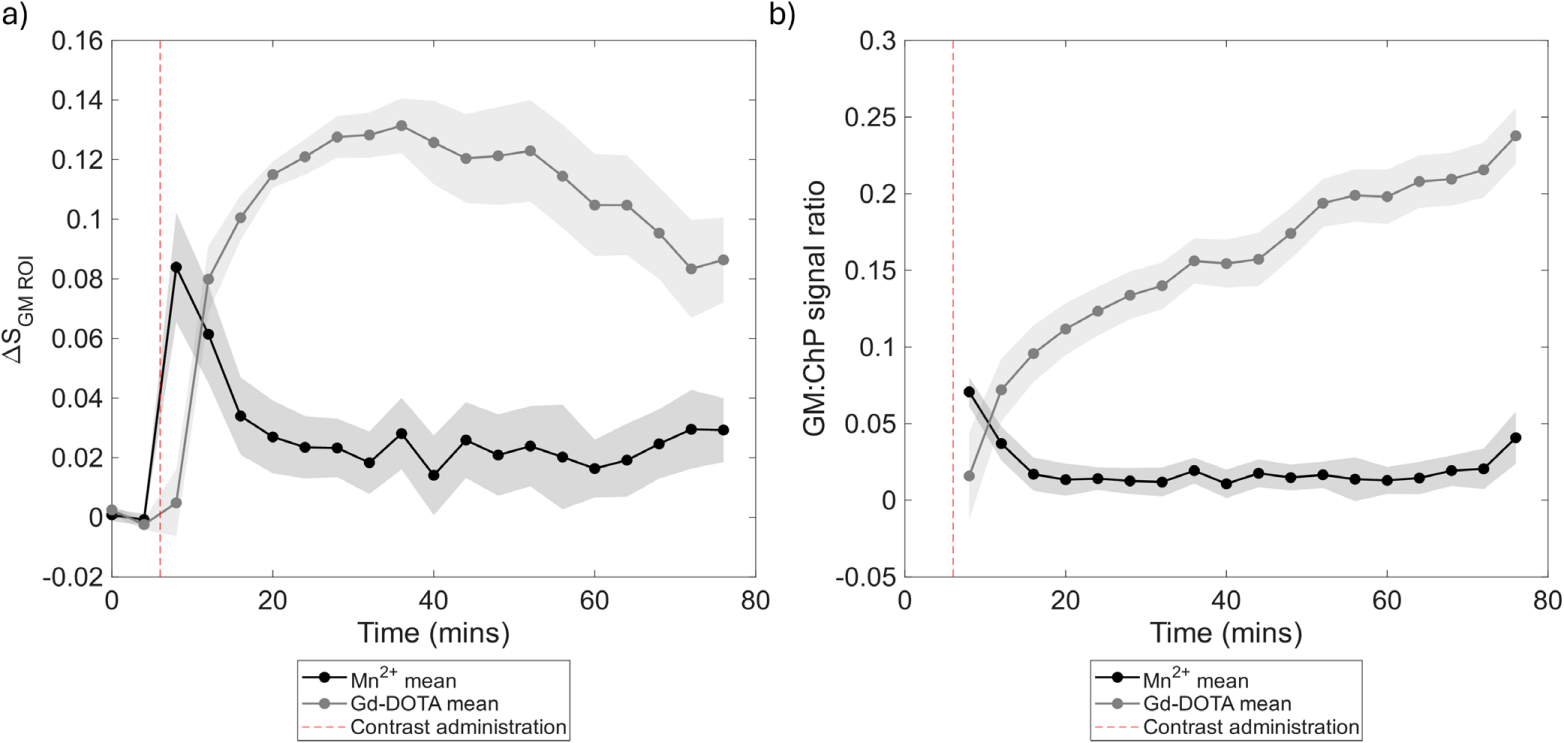
Heavily T2‐weighted FLAIR dynamic contrast‐enhanced MRI data at the cortical gray matter (GM) following intravenous administration of either Mn^2+^ (black) or Gd‐DOTA (gray). (a) Signal enhancement over time in GM. (b) GM:ChP signal ratio over time, after intravenous contrast administration (*t* ≥ 8 min). Mean values shown alongside SEM (*n* = 6 Mn^2+^, *n* = 6 Gd‐DOTA).

Post hoc assessment revealed that cortical GM signal enhancement kinetics differed significantly between contrast agents over time (*F*(19,200) = 4.67, *p* = 7.52 × 10^−9^). Consistent with this, Gd‐DOTA produced a significant, 3× greater GM enhancement than Mn^2+^ at both 16 min (*p* = 0.0049) and 76 min (*p* = 0.033, Figure 4a). To quantify relative tracer distributions, GM:ChP signal ratios were calculated (Figure 4b). These ratios differed significantly between tracers over time (*F*(17,180) = 10.27, *p* = 7.83 × 10^−19^). Ratios were significantly higher—∼six‐fold—for Gd‐DOTA compared with Mn^2+^ at 16 min (*p* = 0.00471) and 76 min (*p* = 1.67 × 10^−5^), indicating more widespread parenchymal enhancement with Gd‐DOTA, whereas Mn^2+^ signal remained comparatively ChP‐dominant.

## 4 Discussion

The ChP is a specialized interface regulating solute exchange between the systemic circulation and the brain, thereby maintaining CNS homeostasis. Yet, in vivo characterization of ChP transport dynamics remains technically challenging. These difficulties arise from its deep intraventricular position and its highly arboreal architecture enveloped by the CSF it secretes and regulates. In this study, we sought to noninvasively resolve the in vivo acute‐phase kinetics of tracer delivery and clearance at the ChP–LV‐CSF interface following intravenous (IV) administration.

Previous MRI approaches examining BCSFB permeability have largely inferred ChP function indirectly through postcontrast enhancement within the ventricular CSF, rather than investigating the uptake/clearance kinetics of the ChP compartment itself. Prior studies using GBCAs employed hT2w‐FLAIR MR cisternography to visualize CSF enhancement as a surrogate of barrier permeability [8, 9]. While these 3D acquisitions provided volumetric coverage, their relatively coarse spatial resolution (∼0.3 × 0.3 mm in‐plane) and the long acquisition time per dataset (∼16 min) limited both temporal and spatial precision [8, 9]. As such, ChP‐specific signal changes are not reported in these earlier studies.

MEMRI has offered an alternative means of probing ChP–CSF physiology, as demonstrated by Aoki et al. [12], who observed Mn^2+^ accumulation within the ChP approximately 10 min after systemic administration, followed by subsequent ventricular and periventricular enhancement. Despite this key finding, the role of the ChP in Mn^2+^ uptake has received limited attention, as MEMRI has more commonly been used to map neuronal activity and calcium‐dependent uptake within brain parenchyma, and at a timescale covering hours to days [12, 17, 26].

The current hT2w‐FLAIR approach overcomes these limitations by combining high in‐plane spatial resolution (78 × 78 μm), 4‐min temporal sampling, and CSF‐nulled contrast optimized for ChP visibility. This configuration allows tracer kinetics to be resolved both within the ChP during the acute delivery phase and the subsequent transfer into ventricular CSF. Resolving dynamic signal changes within the ChP itself, rather than inferring ChP activity from bulk ventricular enhancement, provides a more direct assessment of BCSFB transport than previous cisternography or conventional MEMRI studies [6, 8, 9, 12, 17].

We first administered Mn^2+^, as a proxy for Ca^2+^, to investigate tracer transport. From the images and video (Video S1, Figure 1), there is a striking selectivity for the ChP tissue, compared to the surrounding brain tissue, as evidenced by consistently low relative GM enhancement (Figure 4b). This is in line with the previous autoradiography and other MEMRI experiments, where ChP/CSF enhancement is seen prior to any parenchymal enhancement [1, 12, 17].

The absence of detectable differences in AT or TTP between the ChP and LV‐CSF indicates that transfer from the vasculature to the CSF via the ChP occurs rapidly, without a measurable temporal delay at the resolution used here. Instead, the observed inter‐compartmental differences emerge in the subsequent kinetics, reflected in ingress rates, transient retention, and total tracer accumulation.

These kinetic features are consistent with uptake and transient retention of Mn^2+^ within the ChP ROI—which contains epithelial, stromal, and vascular fractions—and highlight differences in the ChP versus CSF compartmental transport mechanisms. First, stromal Mn^2+^ access is achieved through the fenestrated capillary endothelium where there is passive filtration and diffusion of small solutes into the stromal space [2, 27, 28, 29]. Then, epithelial Mn^2+^ basolateral uptake, subsequent intracellular retention and buffering, and regulated efflux collectively create a transient reservoir at the ChP before redistribution to the ventricular CSF [2, 27, 28, 29]. The steeper apparent clearance from the ChP, compared to the LV‐CSF, likely reflects both onward transfer into the CSF and pump‐mediated reabsorption into the systemic circulation once local saturation is reached [30]. By comparison, transfer of solutes across the ChP epithelium into the LV‐CSF is rate‐limited by various transcellular transport processes, ranging from transporters to vesicles [2, 27, 28, 29]. Taken together, our findings suggest that this regulatory machinery at the BCSFB may be visualized noninvasively using Mn^2+^ as a proxy, probing a regulatory role of the ChP that has remained largely unexplored in vivo.

Conversely, the relatively stable LV‐CSF signal likely reflects ongoing ChP‐driven tracer entry and the physical movement of Mn^2+^‐containing CSF around the ventricular system, as well as CSF moving out of the 2D imaging slice, which causes signal to be lost through the imaging plane.

Using an identical imaging protocol, we also evaluated Gd‐DOTA, as GBCAs have been employed in prior MRI studies of CSF tracer ingress [6]. In pilot experiments, a GBCA dose matching the Mn^2+^ molarity produced substantially weaker signal enhancement (Smax and AUC, see Video S1). Achieving comparable enhancement therefore required a markedly higher Gd‐DOTA concentration, reflecting both its lower relaxivity when chelated [31], and its comparatively limited apparent specificity for ChP uptake and retention. Even at eight‐fold higher molarity, tracer distribution diverged early after administration (*t* ≥ 16 min) and remained distinct throughout the experiment: Gd‐DOTA produced diffuse parenchymal enhancement (Figures 1a,b and 4a,b), whereas Mn^2+^ showed stronger and spatially selective ChP enhancement with minimal cortical GM signal. This persistent divergence indicates that the ChP acts as a preferential sink for Mn^2+^ relative to the surrounding parenchyma. Matching the peak enhancement reduces the likelihood that the difference in kinetic profiles simply reflect insufficient signal generation. Mn^2+^ is a substantially smaller tracer than Gd‐DOTA, which may influence passive diffusion, extracellular distribution, and access to CSF spaces; however, molecular weight alone is unlikely to explain the divergent kinetics. Instead, the dominant distinction is likely tracer‐specific biological handling [13, 22, 27].

When examining tracer‐dependent differences in kinetics at the ChP‐LV‐CSF locus, the most prominent distinctions were the markedly shorter plateau duration and faster clearance rate of Gd‐DOTA compared with Mn^2+^. The ChP is known to exhibit a perfusion rate far exceeding that of the brain parenchyma [4, 24, 32], so both tracers would be expected to reach the ChP capillaries rapidly after administration. Given that our ChP ROI captures both epithelial and microvascular signal, the earliest enhancement likely reflects tracer delivery and clearance within the vascular compartment. Accordingly, both Mn^2+^ and Gd‐DOTA would be expected to exhibit similar kinetics. However, only Mn^2+^ exhibits a sustained ∼14‐min plateau, whereas Gd‐DOTA signal enhancement declines immediately after peaking. This divergence indicates that the prolonged Mn^2+^ signal is reflective of transient epithelial retention, while Gd‐DOTA, which remains predominantly extracellular, clears as soon as vascular washout begins.

This behavior can be accounted for by an absence of intracellular sequestration mechanisms for Gd chelates at the ChP epithelium [6, 8, 9, 10], in contrast to Mn^2+^, which can enter and be homeostatically buffered within epithelial cells [2, 27, 28, 29, 30]. These tracer profiles are also consistent with known systemic pharmacokinetics. Mn^2+^—in the formulation used here—has been shown to undergo rapid plasma clearance (half‐life ∼1.5 min in pigs [20]), consistent with fast redistribution from the circulation into metabolically active organs [33, 34]. In contrast, Gd‐DOTA behaves as an extracellular agent with a plasma elimination half‐life of ∼1 h in rats and predominant renal clearance [35]. Accordingly, the more rapid decline in Gd‐DOTA signal within the combined vascular–epithelial ChP ROI may partly reflect extracellular, vascular washout, whereas the more sustained Mn‐associated signal is more consistent with tissue uptake and retention rather than prolonged vascular residence. A similar pattern is detected in the transient GM signal following Mn^2+^ administration (Figure 4a), which peaks at ∼8 min and declines rapidly thereafter, consistent with rapid perfusion‐driven delivery followed by systemic clearance and limited parenchymal retention relative to ChP uptake. Critically, this indicates that any early vascular/perfusion‐related Mn^2+^ contributions are short‐lived in tissue lacking ChP‐specific uptake or sequestration mechanisms. Therefore, although the ChP ROI represents a mixed vascular–stromal–epithelial compartment and early post‐IV enhancement may include residual microvascular signal, the sustained ChP plateau and divergence from the rapidly decaying GM profile argue against the ChP kinetics being dominated by vascular signal alone.

The most pronounced differences within the LV‐CSF compartment were observed in ΔAT and TTP. After accounting for ChP arrival delays, these parameters were approximately eight‐ and six‐fold longer with Gd‐DOTA, respectively. These findings are in line with the less favorable transfer of Gd‐DOTA from the ChP into the LV‐CSF, compared with Mn^2+^. Consistent with the group‐averaged kinetic curves, the onset of ChP clearance occurred roughly 4 min before any detectable LV‐CSF enhancement. This temporal dissociation suggests that the dominant clearance pathway for Gd‐DOTA at the ChP is venous reabsorption rather than trans‐epithelial transfer into the CSF, further reinforcing the mechanistic distinction between the two tracers.

The current kinetic modeling should be regarded as semi‐quantitative, as no validated framework yet captures the complex, nonlinear transport processes underpinning in vivo ion homeostasis at the BCSFB [15]. Conventional compartmental models based on passive diffusion or bulk flow (e.g., Tofts or Patlak) are poorly suited to systems dominated by active and facilitated transport [36]. Avoiding speculative assumptions, the semi‐quantitative approach applied here enables robust comparison of regional tracer dynamics without over‐interpretation of the biological underpinnings of the fitted rate constants.

We also prioritized optimizing the sequence for sensitivity to CSF‐space delivery of contrast agents over direct vascular quantification. The long‐TE, hT2w, RARE readout, combined with a FLAIR preparation, suppresses baseline CSF signal thereby improving ChP delineation and sharpening the borders of CSF‐containing compartments. This improves our ability to detect postcontrast signal changes within the ventricular CSF, as any incoming tracer produces high relative contrast against the suppressed baseline. By design, the acquisition also markedly attenuates intravascular signal, limiting sensitivity to arterial input dynamics, and instead favoring reliable detection of Mn^2+^ transfer from the ChP into the CSF. Consequently—and together with a conservative averaging scheme to ensure high SNR—temporal resolution was limited to 4 min per scan. Nonetheless, this approach provided sufficient sensitivity to detect subtle CSF‐space kinetic features. Future implementation of accelerated or 3D T1‐weighted readouts (e.g., MPRAGE) could increase temporal sampling, as well as permitting both whole‐ventricular and whole‐brain coverage, which enables more mechanistic compartmental modeling of tracer kinetics across the ventricular system, including the 3rd and 4th ventricles.

Mn^2+^ is both a T1‐ and T2‐shortening contrast agent and so, competing T2 effects must be considered when interpreting hT2w‐FLAIR signal changes. At sufficiently high local Mn^2+^ concentrations, T2/T2* shortening could partially offset T1‐driven signal enhancement, particularly within the ChP, where Mn^2+^ accumulation is expected. Therefore, it is possible that within the ChP ROI, the peak signal is underestimated, and the postpeak decline may represent a combination of Mn^2+^ egress/redistribution and concentration‐dependent T2 attenuation. However, prior work has demonstrated that strong T2 weighting in hT2w‐FLAIR does not abolish contrast‐agent–induced signal changes driven by T1 shortening, while the long T2 of CSF preserves signal at extended echo times. Experimental work has also shown that this approach provides higher sensitivity to nM‐μM contrast‐agent concentrations than conventional T1‐weighted imaging [8, 37]. Together, these sequence features were ideal for our primary aim of determining the spatiotemporal patterns of Mn^2+^ delivery to the CSF through regulated BCSFB transport.

To conclude, this work demonstrates that a FLAIR‐based MEMRI approach can sensitively capture the sequential delivery of Mn^2+^ across the ChP, providing a noninvasive surrogate for Ca^2+^ transport into the CNS. With the optimized parameters used here, we could more reliably resolve and quantify signal contributions from the ChP and LV‐CSF and determine how tracer properties shape BCSFB imaging outcomes. Mn^2+^ therefore provides a probe of ChP transport and offers a platform for investigating molecular regulation of ion exchange at the blood–CSF interface, including pharmacological modulation of ChP transport pathways and pathological contexts such as Alzheimer's disease, where calcium dysregulation is a recognized hallmark [38].

## Funding

This work was supported by Wellcome Trust (212937/Z/18/Z, 223808/Z/21/Z, 225345/Z/22/Z), Parkinson's UK (SFEL‐19‐02, DAA‐25B‐K‐25‐02), Alzheimer's Society (AS‐DRL‐24‐003), Cancer Research UK (190133), Rosetrees Trust, John Black Charitable Foundation (A2200), Brain Tumour Charity (QfC_2018_10387), British Heart Foundation (FS/15/33/31608, FS/SBSRF/21/31020, PG/24/11864, RM/17/1/33377), Medical Research Council (MR/R026416/1), British Council (31BX21DSKV), UCL Birkbeck MRC DTP, and the AstronauTx Ltd. and Eli Lilly & Company.

## Conflicts of Interest

The authors declare no conflicts of interest.

## Supporting information


**Figure S1:** MRI protocol and examples from a representative mouse (*n* = 1): (a) 3D anatomical scan example images from heavily T2‐weighted (hT2w) 3D RARE scan to identify ChP location. (b) hT2w‐FLAIR DCE MRI baseline vs. postcontrast agent images using Mn^2+^. (c) ROI selection from postcontrast (*t* = 8 min) image, for the choroid plexus (ChP, red), lateral ventricular cerebrospinal fluid (LV‐CSF, blue), and cortical gray matter (GM, green).
**Figure S2:** Pilot datasets used to determine primary comparison outputs and dosing. (a) Intravenous Mn^2+^ pilot, monitored over an extended duration of 180 min (*n* = 1). (b) Low‐dose Gd‐DTPA ([11.25 mM], *n* = 1). (c) High‐dose Gd‐DOTA ([90 mM], *n* = 1)—used in final study.

**Data S1:** Supplementary Methods.

**Table S1:** Comparison of tracer kinetics between ChP and LV CSF regions for Mn and Gd‐DOTA studies (*n* = 6 Mn^2+^ paired, *n* = 6 Gd‐DOTA paired).

**Table S2:** Comparison of Mn vs. Gd‐DOTA tracer kinetics across ChP and LV CSF parameters (*n* = 6 vs. 6, unpaired).

**Video S1:** Side‐by‐side comparison of contrast agent kinetics in the mouse brain: Mn^2+^ and GBCAs.

## Data Availability

Upon publication, the MRI data will be made freely available to download from the UCL data repository (https://rdr.ucl.ac.uk/authors/Jack_Wells/6768476).
