# Supplementary material for: Using Manganese-Enhanced DCE-MRI to Assess Blood-Cerebrospinal Fluid Barrier Transport
Source: Magn Reson Med. Author manuscript; Available in PMC 2026 Nov 13. (PMC13527285; doi:10.1002/mrm.70531)
Supplement: Supplementary file 2 [file EMS217521-supplement-Supplementary_Table_1.docx]

| Parameter | Test | ChP mean | ChP SEM | LV CSF mean | LV CSF SEM | Raw p | FDR p |
| --- | --- | --- | --- | --- | --- | --- | --- |
| **Mn^2+^** | | | | | | | |
| AT (min) | t-test | 0.13822 | 0.13822 | 0.78106 | 0.37087 | 0.19379 | 0.19379 |
| rise rate (S.min^-1^) | Wilcoxon | 0.56881 | 0.17293 | 0.10905 | 0.07916 | 0.03125 | 0.03125 |
| TTP (min) | Wilcoxon | 5.60070 | 0.86349 | 7.91450 | 2.08880 | 0.21875 | 0.21875 |
| Smax (A.U.) | t-test | 1.51520 | 0.21054 | 0.27029 | 0.04712 | 0.00281 | 0.00281 |
| clearance rate (S.min^-1^) | t-test | 0.00962 | 0.00235 | 0.00822 | 0.00306 | 0.57858 | 0.57858 |
| AUC ((ΔS_ROI_)/S_GM_).min) | t-test | 91.10100 | 15.16300 | 15.38800 | 2.51610 | 0.00522 | 0.00522 |
| plateau duration (min) | t-test | 14.08500 | 1.74950 | 23.57100 | 8.41070 | 0.30762 | 0.57858 |
| **Gd-DOTA** | | | | | | | |
| AT (min) | t-test | 0.00000 | 0.00000 | 4.92320 | 2.09680 | 0.07117 | 0.07117 |
| rise rate (S.min^-1^) | Wilcoxon | 0.12999 | 0.03298 | 0.01998 | 0.01364 | 0.06250 | 0.06250 |
| TTP (min) | Wilcoxon | 6.00000 | 0.00000 | 19.36400 | 3.50950 | 0.06250 | 0.06250 |
| Smax (A.U.) | t-test | 1.26310 | 0.15709 | 0.28120 | 0.09655 | 0.00023 | 0.00023 |
| clearance rate (S.min^-1^) | t-test | 0.02133 | 0.00221 | 0.03187 | 0.01268 | 0.47462 | 0.71238 |
| AUC ((ΔS_ROI_)/S_GM_).min) | t-test | 56.12900 | 10.11100 | 11.60900 | 4.31300 | 0.00166 | 0.00166 |
| plateau duration (min) | t-test | 6.30870 | 2.56190 | 8.17370 | 3.27550 | 0.71238 | 0.71238 |

**Table S1.** *Comparison of tracer kinetics between ChP and LV CSF regions for Mn and Gd-DOTA studies (n = 6 Mn^2+^ paired, n = 6 Gd-DOTA paired).*

Group means and standard errors of the mean (SEM) are shown for Mn^2+^ and Gd-DOTA datasets across multiple kinetic parameters, comparing ChP and LV CSF regions for each tracer. Paired comparisons were conducted within subjects for each parameter, using paired t-tests when both regional datasets passed normality (Shapiro-Wilk, p > 0.05), or Wilcoxon signed-rank tests when normality was not satisfied. Primary hypothesis variables are reported with raw p-values (rows with white background). Non-primary parameters were subjected to Benjamini–Hochberg false discovery rate (FDR) correction and are shaded in light grey. For secondary parameters, adjusted p-values are shown; where no FDR correction was applied, the FDR p-value equals the raw p-value. Statistically significant comparisons (p < 0.05) are indicated in red text.
