# Supplementary material for: Using Manganese-Enhanced DCE-MRI to Assess Blood-Cerebrospinal Fluid Barrier Transport
Source: Magn Reson Med. Author manuscript; Available in PMC 2026 Nov 13. (PMC13527285; doi:10.1002/mrm.70531)
Supplement: Supplementary file 3 [file EMS217521-supplement-Supplementary_Table_2.docx]

| Parameter | Test | Mn^2+^ mean | Mn^2+^ SEM | Gd-DOTA mean | Gd-DOTA SEM | Raw p | FDR p |
| --- | --- | --- | --- | --- | --- | --- | --- |
| ChP AT (min) | MW | 0.13822 | 0.13822 | 0.00000 | 0.00000 | 0.58225 | 0.71958 |
| ChP rise rate (S.min^-1^) | Welch | 0.56881 | 0.17293 | 0.12999 | 0.03297 | 0.05171 | 0.29748 |
| ChP TTP (min) | MW | 5.60070 | 0.86349 | 6.00000 | 0.00000 | 0.06494 | 0.29748 |
| ChP Smax (A.U.) | Welch | 1.51520 | 0.21054 | 1.26310 | 0.15709 | 0.36169 | 0.66309 |
| ChP plateau duration (min) | Welch | 14.08500 | 1.74950 | 6.30870 | 2.56190 | 0.03395 | 0.03395 |
| ChP decay rate (S.min^-1^) | Welch | 0.00962 | 0.00235 | 0.02133 | 0.00221 | 0.00465 | 0.00465 |
| ChP AUC ((ΔS_ROI_)/S_GM_).min) | Welch | 91.10100 | 15.16300 | 56.12900 | 10.11100 | 0.08827 | 0.29748 |
| LV CSF AT (min) | MW | 0.78106 | 0.37087 | 4.92320 | 2.09680 | 0.24026 | 0.24026 |
| LV CSF rise rate (S.min^-1^) | MW | 0.10905 | 0.07916 | 0.01998 | 0.01364 | 0.58874 | 0.71958 |
| LV CSF TTP (min) | Welch | 7.91450 | 2.08880 | 19.36400 | 3.50950 | 0.02267 | 0.02267 |
| LV CSF Smax (A.U.) | Welch | 0.27029 | 0.04712 | 0.28120 | 0.09655 | 0.92189 | 0.99062 |
| LV CSF plateau duration (min) | Welch | 23.57100 | 8.41070 | 8.17370 | 3.27550 | 0.13522 | 0.29748 |
| LV CSF decay rate (S.min^-1^) | Welch | 0.00822 | 0.00306 | 0.03187 | 0.01269 | 0.12351 | 0.29748 |
| LV CSF AUC ((ΔS_ROI_)/S_GM_).min) | Welch | 15.38800 | 2.51610 | 11.60900 | 4.31300 | 0.47074 | 0.71958 |
| ΔSmax (A.U.) | Welch | 0.20168 | 0.04334 | 0.20084 | 0.05467 | 0.99062 | 0.99062 |
| ΔAT (min) | Welch | 0.64284 | 0.31426 | -4.92320 | 2.09680 | 0.04485 | 0.04485 |
| ΔTTP (min) | Welch | 2.31390 | 1.60710 | 13.36400 | 3.50950 | 0.02421 | 0.02421 |

**Table S2.** *Comparison of Mn vs Gd-DOTA tracer kinetics across ChP and LV CSF parameters (n = 6 vs 6, unpaired).*

Group means and standard errors of the mean (SEM) are shown for Mn and Gd-DOTA datasets across multiple kinetic parameters. Unpaired comparisons were performed between Mn^2+^ and Gd-DOTA groups for each parameter, using Welch’s t-test if both datasets passed normality (Shapiro–Wilk, *p* > 0.05) or Mann–Whitney (MW) U test otherwise. Primary hypothesis variables are reported with raw *p*-values and are displayed with a white background. Non-primary outputs underwent Benjamini–Hochberg false discovery rate (FDR) correction and are shaded in light grey, and FDR-adjusted *p*-values are shown. Where no FDR correction was applied, the adjusted *p*-value equals the raw *p*-value. Red text is used in instances where statistically significant differences were identified (p < 0.05).
