# Supplementary material for: Using Manganese‐Enhanced DCE‐MRI to Assess Blood‐Cerebrospinal Fluid Barrier Transport
Source: Magn Reson Med. 2026 Jul 26;96(5):2083–91. doi: 10.1002/mrm.70531 (PMC13527285; doi:10.1002/mrm.70531)
Supplement: Supplementary file 1 [file MRM-96-2083-s001.docx]

**Supplementary Figures and Video**

[Video S1 – uploaded separately]

**Video S1 -** *Side-by-side comparison of contrast agent kinetics in the mouse brain: Mn^2+^ and GBCAs.*

This video presents synchronized axial slices from three mice imaged over 76 minutes, illustrating the dynamic distribution of intravenously administered contrast agents within the brain. Each panel displays a different experimental condition in a representative mouse (left: 11.25 mM Mn²⁺, middle: 11.25 mM Gd-DTPA (from pilot study), right: 90 mM Gd-DOTA).

All datasets were aligned spatially and intensity-scaled uniformly to facilitate direct visual comparison. A time stamp is shown in the top right corner, beginning at 0 minutes and increasing in 4-minute intervals, capturing the progression of contrast agent uptake and clearance over time.

**Figure S1 -** MRI protocol and examples from a representative mouse (n = 1): a) 3D anatomical scan example images from heavily T2-weighted (hT2w) 3D RARE scan to identify ChP location. b) hT2w-FLAIR DCE MRI baseline vs post-contrast agent images using Mn^2+^. c) ROI selection from post-contrast (t = 8 min) image, for the choroid plexus (ChP, red), lateral ventricular cerebrospinal fluid (LV-CSF, blue), and cortical grey matter (GM, green).

**Figure S2** – Pilot datasets used to determine primary comparison outputs and dosing. a) Intravenous Mn^2+^ pilot, monitored over an extended duration of 180 mins (n = 1). b) Low-dose Gd-DTPA ([11.25mM], n = 1). c) High-dose Gd-DOTA ([90 mM], n = 1) – used in final study.
