# Supplementary material for: Using Manganese‐Enhanced DCE‐MRI to Assess Blood‐Cerebrospinal Fluid Barrier Transport
Source: Magn Reson Med. 2026 Jul 26;96(5):2083–91. doi: 10.1002/mrm.70531 (PMC13527285; doi:10.1002/mrm.70531)
Supplement: Supplementary file 2 [file MRM-96-2083-s002.docx]

**Supplementary Methods**

Modelling

In order to obtain spatiotemporal parameters to describe signal changes within the ChP and LV-CSF compartments, as well as derive metrics that describe tracer movement between the compartments, we applied a semi-quantitative model (Matlab R2025a).

The model equation is:

[ΔS_ROI_(t)]/S_GM_ =
 0,	if t < AT
 Smax * [1 - exp(-k_rise_ * (t - AT))] / [1 - exp(-k_rise_ * T_rise_)], if AT ≤ t < AT + T_rise_
 Smax,	if AT + T_rise_ ≤ t < AT + T_rise_ + T_plateau_
 Smax * exp(-k_clear_ * [t - (AT + T_rise_ + T_plateau_)]),	if t ≥ AT + T_rise_ + T_plateau_

This piecewise formulation captures the initial zero baseline, a monoexponential rise toward a plateau, a constant plateau segment, and an exponential washout. A regularisation term was included during fitting to promote a smooth and physiologically plausible transition from the rising phase to the plateau. Specifically, a lambda-weighted penalty (λ) was added to the objective function, based on the slope of the signal at 90% of the rise duration. This penalisation discouraged the model from misclassifying early plateau points as part of the rising phase, and thereby served to improve robustness of fits without enforcing a hard threshold.

Parameters were estimated using nonlinear least squares with a multistart strategy (100 initialisations) implemented in MATLAB.

Outcomes and statistical comparison

Primary outcome parameters were defined *a priori* based on pilot data and their relevance to contrast-agent delivery dynamics. For ChP vs LV-CSF comparisons, pilot Mn^2+^ studies (Figure S2) showed faster tracer delivery, shorter TTP, and higher AUC and Smax in the ChP. Accordingly, rise rate, TTP, AUC, and Smax were designated as primary outcomes, alongside AT which reflected the expected progression of tracer transit from ChP to LV-CSF. Remaining parameters (e.g., clearance rate, plateau duration) were considered non-primary.

For Mn²⁺ vs Gd-DOTA comparisons, primary outcomes were defined separately for each compartment and for inter-compartment metrics, based on pilot Gd-DOTA data (Figure S2). In the ChP, clearance rate and plateau duration were selected to assess tracer retention and clearance. In the LV-CSF, AT, rise rate, TTP, and derived ΔAT and ΔTTP were prioritised to capture temporal delivery offsets between compartments.

Group mean signal time courses (±SEM) were plotted for visual comparison. Statistical analyses were performed in MATLAB R2025a. Normality was assessed using the Shapiro–Wilk test; paired t-tests or Wilcoxon signed-rank tests were applied for within-subject comparisons, e.g. ChP vs LV-CSF; and Welch’s t-tests or Mann–Whitney U tests for between-group comparisons, e.g. Mn²⁺ vs Gd-DOTA, as appropriate.

Two-way repeated-measures ANOVA was used to assess contrast agent × time effects, with contrast agent as the between-subject factor and time as the within-subject factor. Predefined primary hypotheses were tested without correction; *non-primary* comparisons were adjusted using the Benjamini–Hochberg FDR method. Full statistics are reported in Tables S1 and S2.

For brevity, the Results section reports only the relevant test statistics (F values, degrees of freedom, and p-values).
